# Development of an Online Training Course for Government Employees to Support Families With a Child With Profound Intellectual and Multiple Disabilities

**DOI:** 10.1111/jar.70039

**Published:** 2025-03-25

**Authors:** Siméon T. A. Lahaije, Jorien Luijkx, Aly Waninge, Annette A. J. van der Putten

**Affiliations:** ^1^ Faculty of Behavioural and Social Sciences, Inclusive and Special Needs Education University of Groningen Groningen the Netherlands; ^2^ Faculty of Healthy Ageing, Allied Health Care and Nursing Hanze University of Applied Sciences Groningen the Netherlands; ^3^ Department of Health Psychology University Medical Center Groningen Groningen the Netherlands

**Keywords:** design thinking, disability, family support, family well‐being, profound intellectual and multiple disabilities, service delivery

## Abstract

**Background:**

Families with a child with profound intellectual and multiple disabilities have to manage the pervasive support needs of their child, which is a complex task. By properly supporting them, we can ensure these families live a positive and meaningful life.

**Method:**

A product to support these families was developed using the method of Design Thinking, which included parents filling out three different questionnaires.

**Results:**

It was found that parents preferred to be supported in strengthening their resilience and care capacity, specifically by improving the communication and (mutual) understanding between them and municipal government employees. An online training course for government employees was subsequently developed.

**Conclusions:**

This study has led to the development of a product that can support families with a child with profound intellectual and multiple disabilities according to their needs and preferences. This study has also provided insights into how these parents prefer to be supported.

**Summary**

- Families with a child with profound intellectual and multiple disabilities have to manage the pervasive support needs of their child.
- To support these families, we developed a product using the method of Design Thinking.
- This product can support families with a child with profound intellectual and multiple disabilities to live a positive and meaningful life.
- This study has also provided insights into the needs, preferences and wishes of families with a child with profound intellectual and multiple disabilities regarding their support.

## 1 Introduction

Supporting persons with a disability within a family context has become a core theme in disability research, practice, and policy, emphasising family‐centred services and research (Samuel et al. 2012; Singer and Wang 2014; Wang and Brown 2009). This is due to the beliefs that family members are the best helpers and advocates of the person with a disability, know their well‐being best, and play a constant role in their lives (Wang and Brown 2009). Moreover, all family members affect each other (White et al. 2018), and well‐functioning families make meaningful contributions to society (Burton‐Smith et al. 2009; Samuel et al. 2012). Support within a family context fits with the contemporary view on disability and families, which focuses on empowering families by focusing on their strengths, adapting and transforming all levels of the socio‐ecological environment to accommodate families, and enabling and supporting their self‐determination in deciding the well‐being they wish to experience (Samuel et al. 2012; Thompson et al. 2017; Turnbull and Turnbull 2002). Families with a child with profound intellectual and multiple disabilities (also known as persons with complex [care] needs, severe disabilities, or profound and multiple learning difficulties) should be supported similarly.

Persons with profound intellectual and multiple disabilities share two defining characteristics, a profound intellectual disability and a profound motor disability (Nakken and Vlaskamp 2007; Vlaskamp 2020). The profound intellectual disability is defined as an IQ standard score < 20–25 points (Schalock et al. 2021) but can only be estimated, not measured (Nakken and Vlaskamp 2007; Van der Putten et al. 2017; Vlaskamp 2020). Due to the profound motor disability, persons with profound intellectual and multiple disabilities require physical assistance or a (powered) wheelchair for moving, and may be limited in the functional use of their hands, arms, and legs (Nakken and Vlaskamp 2007; Van der Putten et al. 2017). Additionally, persons with profound intellectual and multiple disabilities frequently experience sensory impairments (Nakken and Vlaskamp 2007). They often need support for their health, such as for obstipation, gastroesophageal reflux disease, epilepsy, and scoliosis (Nakken and Vlaskamp 2007; Van Timmeren et al. 2017), are frequently hospitalised (Seliner et al. 2016), and often use multiple medicines simultaneously (Van der Heide et al. 2009; Nakken and Vlaskamp 2007). Their communication, consisting of sounds, bodily movements, and physiological signals (e.g., perspiration and pupillary responses), is pre‐linguistic, atypical, and idiosyncratic, and mostly elicited by others (Dhondt et al. 2023; Hostyn et al. 2010; Nakken and Vlaskamp 2007; Van der Putten et al. 2017). Augmentative and alternative communication aids (e.g., pictures and speech‐communicating devices) may facilitate communication (Flink 2022). Additionally, they need support for personal development, leisure activities and societal participation. Altogether, their support needs can be classified as pervasive (Schalock et al. 2021).

It is most often family members who are managing these support needs, often lifelong, even when the person with profound intellectual and multiple disabilities no longer lives at home (Kruithof et al. 2020; Vlaskamp 2020; Wang and Brown 2009). Managing and providing this support is a complex task that requires significant commitment and investment in terms of time and energy (Geuze et al. 2022), and consists not only of caring for the person with profound intellectual and multiple disabilities on a daily basis, but also of arranging and managing a complex and often changing professional support network (Geuze and Goossensen 2021; Kruithof et al. 2020; Nakken and Vlaskamp 2007), as well as acquiring and managing additional support (e.g., government support, peer support) from various domains. Due to the intensity and complexity of the support, parents of a child with profound intellectual and multiple disabilities have less leisure time compared to parents of typically developing children (Luijkx et al. 2017), mention that maintaining the well‐being of their family is a complex and continuous task (Geuze et al. 2022), and need a variety of resources, such as respite care, to maintain their well‐being (Lahaije et al. 2023a). On average, families with a child with profound intellectual and multiple disabilities experience a positive family resilience (Lahaije et al. 2024), as well as a positive family quality of life (FQOL), though their experience of their emotional well‐being (e.g., having time for yourself, having support to relieve stress) is least positive (Lahaije et al. 2023b). By properly supporting families with a child with profound intellectual and multiple disabilities, we can ensure they (continue to) experience a positive well‐being.

Following the central themes in the contemporary view on disability and families, support should be developed with a focus on families' strengths, on empowering them, and on adapting the socio‐ecological environment (Samuel et al. 2012; Thompson et al. 2017; Turnbull and Turnbull 2002). Furthermore, in order for support to be appropriate and applicable, it should be tuned to the needs and preferences of the end‐users, those who are supported. To ensure this, support should not be developed top‐down, rather bottom‐up, in that the end‐users should be engaged in the design process itself. By engaging with the end‐users, the quality of the outcomes is increased, the outcomes are better accepted, and are more relevant and a better fit with the end‐users' needs (Antonini 2021; Shen et al. 2017; Turnbull et al. 1998). Additionally, engaging families in research empowers them as well (Turnbull et al. 1998). Moreover, as parents are the experts in relation to their child with a disability and their family unit, they should naturally be engaged in the development of a product or service aimed at supporting them (Alsem et al. 2017).

A well‐known method to design support solutions in the form of products or services in which the end‐users are personally involved is Design Thinking. Originating in design disciplines (e.g., architecture), Design Thinking has become a popular method used successfully across various disciplines, including health care, public policy, management, and business, as a user‐centric approach to designing solutions to different types of problems (Dorst 2011; Kumar et al. 2016; Luka 2014). Strengths of Design Thinking include that it is human‐centred, holistic, allows for creative and original solutions, is adaptable to ill‐defined, complex or unknown problems, and applying it requires little to no training (Dam and Siang 2018; Kumar et al. 2016; Luka 2014). Additionally, the framework of the design process is open and flexible, which means it can be shaped and adapted to various circumstances, requirements, and preferences, and allows developers to freely select the most appropriate quantitative and/or qualitative methods (Bakusevych 2021). Moreover, the iterative and flexible approach ensures original solutions that best fit the preferences and needs of the end‐users (Kumar et al. 2016; Luka 2014).

To ensure families with a child with profound intellectual and multiple disabilities experience and maintain the positive well‐being they wish to experience, a product or service to support them should be created by engaging with family members themselves, so as to tune the support to their needs and preferences. The aim of this study is therefore to develop a product to support families with a child with profound intellectual and multiple disabilities through the method of Design Thinking.

## 2 Method and Results

### 2.1 Materials

To develop the product, the method of Design Thinking was used, specifically as described by the Hasso Plattner Institute of Design of Stanford University (Dam and Siang 2018). This method or framework consists of the following five phases: (a) Empathise (engaging with and learning about the end‐users of the product), (b) Define (defining what problem the end‐users want to have a product for), (c) Ideate (generate diverse ideas for possible products and determine with the end‐users which idea should be turned into a product), (d) Prototype (create one of more working prototypes of the product), and (e) Test (test the prototype[s] with the end‐users). This method can be iterative, in that any steps may be repeated in any order, if necessary. Moreover, it is an open framework and can be shaped according to the demands and preferences of the end‐users, developers, circumstances, and the process itself (Bakusevych 2021). For this study, we opted to engage with the end‐users (parents of a child with profound intellectual and multiple disabilities) through online questionnaires, as we expected it would be difficult to find a suitable time to gather a sufficient number of parents to meet simultaneously, while online questionnaires would reach a higher number of participants and would allow participants to participate at their own time and pace. Participants were involved in the Define, Ideate and Test phases, but not in the Empathise phase, as we had learned about the lives and needs of families with a child with profound intellectual and multiple disabilities through previous studies and therefore empathised with them.

### 2.2 Ethics

After independent review, the ethical committee of the department of Pedagogical & Educational Sciences (Ethische Commissie PEDON) granted ethical permission for this study. Participants were informed of the nature and goal of the study, data handling and data privacy by an information brochure. Written informed consent was gathered from all participants. Participants received no financial compensation but were informed of the outcomes of the study.

### 2.3 Recruitment

Participants were recruited in the Netherlands through newsletters and magazines, social media, personal invitations, leaflets, and letters sent out to service‐providing facilities, websites and conferences. The inclusion criteria for the participants were being a Dutch‐speaking parent of a child with profound intellectual and multiple disabilities (profound intellectual disability, that is, estimated IQ of < 25 points and/or developmental age < 24 months, and profound motor disability, that is, level IV or V on the Gross Motor Functioning Classification System [GMFCS; Palisano et al. 1997]) between the ages of 0 and 30 years, from the Netherlands. Participants were asked to complete a questionnaire regarding several characteristics of their child with profound intellectual and multiple disabilities (i.e., indication of the level of the intellectual disability, motor functioning skills, communicative abilities, and additional health problems) by which the researcher could determine if the child had profound intellectual and multiple disabilities and the participant thus met the inclusion criteria. A total of 50 parents met the criteria. A majority of the participants had also participated in other studies in the same project as this study.

### 2.4 Empathise

To empathise with the end‐users and learn about their lives, needs, and which problems they are facing, we re‐familiarised ourselves with the outcomes of our previous studies on FQOL (Lahaije et al. 2023b), support needs (Lahaije et al. 2023a), and family resilience (Lahaije et al. 2024). Moreover, the outcomes of other studies (Gauthier‐Boudreault et al. 2017; Jansen et al. 2013; Kruithof et al. 2022a, 2022b; Zaal‐Schuller et al. 2016) on the needs of these families were considered as well. Based on these outcomes, 15 problems were selected by the first author for parents to rank in order of importance (see Table 2 for an overview of these problems). Considerations for this selection were the feasibility of developing a product or service for each problem and presenting a diverse array of problems from all levels of the socio‐ecological environment.

### 2.5 Define

Determining which problem parents found most important to have a product for was the goal of the Define phase. To this end, all parents who signed up, agreed to participate in this study, and met the inclusion criteria (parent of a child with profound intellectual and multiple disabilities between the ages of 0 and 30 years) were invited to participate in this phase. Reminders were sent out bi‐weekly, up to a maximum of four. Of the 50 parents who were invited, 34 responded. These were six fathers aged 44 to 69 years (M = 52.5, SD = 9.3) and 28 mothers aged 30 to 67 years (M = 47.3, SD = 8.4), all from separate families. Their children were aged 4 to 31 years (M = 13.7, SD = 7.2).

Participants were asked to fill out a short questionnaire to determine which problem they found most important to be supported with. After a short introduction about the set‐up and purpose of the questionnaire participants were asked to provide some background characteristics (e.g., family income, number of family members) of their family and its members to have an overview of the study population (see Table 1 for the overview of the characteristics). Participants were then presented with the 15 selected subjects or problems with a short description in a random order and were asked to rank them based on importance (see Table 2 for an overview of the problems). They could also leave suggestions or remarks, including additional subjects they would find important.

**TABLE 1:** Characteristics of Participants (Familial and Personal) of Define Phase.

Characteristic (family)	Families (*n* = 34)
	*n*
Family type	
Two parent family	29
Single parent family	5
Annual family income	
< € 20,000	2
€ 20,000–€ 65,000	14
> € 65,000	12
Unknown	6
Number of family members	
2	2
3	9
4	14
5	9

Characteristic (person)	Fathers (*n* = 6)	Mothers (*n* = 28)
	*n*	*n*
Highest educational level		
Secondary education	0	2
Intermediate vocational training (MBO [Middelbaar beroepsonderwijs])	1	4
Higher vocational training (HBO [Hoger beroepsonderwijs])	2	14
University (WO [Wetenschappelijk onderwijs])	3	8
Employment		
Full‐time (≥ 35 h)	4	7
Part‐time (0–35 h)	1	19
Unemployed	1	2

**TABLE 2:** Ranking and Total Score per Subject in Define Phase.

Rank position	Subject	Total points
1	Information about applications/welfare programs/administration (what/when/where/how)	59
2	Information about assistive technology (what/when/where/how)	55
3	Finding and improving a balance between care load and care capacity (by strengthening care capacity)	48
4	Transfer support and (parental) knowledge of the child with profound intellectual and multiple disabilities to others	42
5	Strengthening resilience	41
5	(Improve) contact with government officials/health care insurance companies/other organisations	41
7	(Improve) contact with healthcare professionals	37
8	Toys (for leisure and development)	35
9	Information about day care centres/places to stay over/residential facilities	33
9	Being an employer as part of managing a *PGB* (personal budget)	33
11	Palliative care and quality of life	32
12	Having activities together as a family (at home or outside of home)	30
13	Combining work and informal care	17
14	(Improve) contact with peers	4
15	(Improve) contact with family/friends/neighbours/colleagues/others	3

To analyse the outcomes of the questionnaire and determine the overall ranking of the problems, a positional voting system (Llamazares and Peña 2015) with the following scoring rules was conceived. For each participant, the highest ranked problem was assigned five points, the second highest four, the third highest three, the fourth highest two, and the fifth highest one point. Lower ranked problems received no points. For each problem, the total score was calculated as the sum of the points given by the participants.

The results are shown in Table 2, which includes the total amount of points for each problem, with problems ranked by the total score. The suggestions and remarks by the participants did not reveal any additional shared problems. Informational support regarding assistive technology, as well as regarding administrative matters, were rated highest. Conversely, improving contact with either peers or family, friends and so forth were scored lowest and also received a relatively limited number of points. Finding and improving balance (by strengthening care capacity) was ranked highest the most (6 times), while improving contact with peers, and family and friends, and strengthening resilience were never ranked highest. However, strengthening resilience was ranked second highest the most (8 times). Of the different problems regarding communication, improving contact with healthcare professionals and government employees was ranked distinctly higher than improving contact with peers, and family and friends.

### 2.6 Ideate

The goal of this phase was to determine which idea parents preferred for a product for support. Although informational support regarding administrative matters as well as assistive technology were ranked highest in the previous phase (see Table 2), it had (informally) come to our attention that products offering this informational support (as well as a product related to the fourth highest ranked problem, transferring parental knowledge) were already under development in the Netherlands. Therefore, we opted to focus on a combination of the third and fifth highest ranked problems and develop a product to strengthen family resilience and care capacity. Nine diverse solutions or ideas for a product (i.e., different types of products and interventions, focusing on both internal and external factors) were then created by the first author accordingly (see Table 3 for an overview of the ideas).

**TABLE 3:** Ranking and Statistics per Idea in Ideate Phase.

Rank position	Idea	M	SD
1	Communication training for healthcare professionals and/or government employees	7.9	1.5
2	Public awareness campaign on families with a child with profound intellectual and multiple disabilities	7.4	1.8
3	Resilience and care capacity broker	6.8	1.8
4	Physical and mental resilience training	6.3	1.6
4	Outdoor family challenge	6.3	1.7
6	E‐learning resilience	6.2	1.7
7	Training on improving family communication	6.0	2.0
8	Training on dealing with stigma	5.2	1.9
9	Resilience app	5.1	2.3

All participants who had participated in the previous phase were invited to the Ideate phase. Of the 34 invited participants, 25 responded: 4 fathers aged 44 to 69 years (M = 54.8, SD = 10.7), and 21 mothers aged 30 to 59 years (M = 46.7, SD = 8.1). Reminders were sent out bi‐weekly, up to a maximum of two.

Participants were again asked to fill out a short questionnaire, now to determine which idea they found most preferable and should therefore be developed into a product. This questionnaire consisted of a short introduction about the set‐up and purpose of the questionnaire, after which the participants were presented with the nine ideas (including a short description) for a product to support families in strengthening their resilience and care capacity in random order. To determine which idea the participant found best to solve the determined problem, they were asked to rate each idea on a scale from 0 to 10. Participants were also asked what they thought about the idea and if they had any suggestions on how to change or improve it. At the end of the questionnaire, participants were able to leave suggestions or remarks. For each idea, the average score and standard deviation were calculated.

The average scores of each idea are presented in Table 3. Communication training for health care professionals and/or government employees was rated the highest, and an app to strengthen resilience rated the lowest. The two highest‐rated ideas both consist of some form of adapting or transforming the environment to accommodate families. Additionally, several parents explicitly commented that they did not prefer solutions that required an investment on their part, as they stated to be limited in the time they could spare and rather preferred solutions in which others had to take action.

### 2.7 Prototype

Based on the outcomes of the previous phase, an initial prototype of the product was developed in the Prototype phase. The preferred idea for a product was communication training for health care professionals and/or government employees. As we were aware (based on the outcomes of our previous study [Lahaije et al. 2023a]) that parents and families of a child with profound intellectual and multiple disabilities have different relationships and expectations of health care professionals compared to government employees, it was opted for applicability and effectiveness to develop a product for one of these groups. We selected the group of government employees, as we expected them to be considerably less familiar with families with a child with profound intellectual and multiple disabilities and their needs than health care professionals.

In the Netherlands, there are several government programs that relatives of a person with profound intellectual and multiple disabilities can apply for. One of these is the *Wet Maatschappelijke Ondersteuning* (WMO; Social Support Act), by which the municipality provides social support (e.g., assistive technology, domestic help, or respite care) through either customised or standard services for persons who are unable to cope on their own or are unable to fully participate in society (Rijksoverheid 2023). For persons with profound intellectual and multiple disabilities, the WMO can, for example, provide assistive technology, as well as respite care for relatives. While the social support offered through the WMO is not a limited resource, the (conditions for) support and services offered may vary between municipalities. WMO applications are managed by municipal government employees who specialise in these applications (*WMO‐consulenten*), and the focus of the product was therefore aimed at this specific group of government employees.

To better understand the WMO (applications) and the role and duties of government employees, we held informational talks with a parent who engaged with her municipality on this subject, two elected municipal council members, and a government employee who managed WMO applications and who was also a parent of a child with profound intellectual and multiple disabilities. Additionally, we informed ourselves on how government employees in the Netherlands are trained and educated, reacquainted ourselves with what parents had expressed about the WMO in our previous study on the needs of families with a child with profound intellectual and multiple disabilities (Lahaije et al. 2023a), and read a guide on WMO applications (Claassen and Van der Kooij 2022) as well as publications from the Dutch National Ombudsman about the Social Support Act (Jonquière et al. 2023) and from the Association of Netherlands Municipalities on client support (Schoenmakers et al. 2022).

The informal talks, as well as the various publications, provided us with more insight into the organisation of the municipal government, the management of WMO applications, the relevant laws and policies, how government employees were trained (i.e., online training courses or e‐learning [VNG Connect 2024]), what their needs were, and what was important to parents. Based on these insights, the goal of the training course was defined as improving the communication and mutual understanding between relatives of a person with profound intellectual and multiple disabilities and government employees who manage WMO applications. To this end, the first author (with input from the co‐authors) wrote two introductory texts, one about persons with profound intellectual and multiple disabilities and their families, and one about why they applied for government support and what difficulties they encountered. Additionally, the first author (with input from the co‐authors) formulated 10 statements of what would be important to relatives of a person with profound intellectual and multiple disabilities in relation to the management of WMO applications and the communication of the government employees. The texts and statements together formed the prototype of the training course.

### 2.8 Test

For the final phase, all participants who participated in the Ideate phase were invited to test the prototype created in the previous phase, so as to further refine it. Of the 25 participants invited, 21 responded: 4 fathers aged 44 to 69 years (M = 54.8, SD = 10.7) and 17 mothers aged 30 to 59 years (M = 46.9, SD = 8.0). Reminders were sent out bi‐weekly, up to a maximum of two.

Participants were asked to fill out a questionnaire about the texts and statements created in the previous phase. After a short introduction about the set‐up and purpose of the questionnaire, namely to test and refine the prototype, participants were presented with the two introductory texts, one describing persons with profound intellectual and multiple disabilities and their families, and one describing why they applied for government support and what difficulties they encountered. Participants were asked to rate each text's clarity, as well as to what degree they expected each text to lead to a better understanding of their lives, both on a scale from 1 to 5. Next, participants were presented with two sets of five statements regarding the communication of government employees and the services they provide. They were asked to rate each statement on importance on a scale from 1 to 5. Table 4 shows an overview of the statements. Participants could leave suggestions or remarks at the end of the questionnaire. Average scores for the clarity and expected increased understanding of both texts were calculated, as well as the average score and standard deviation for each statement.

**TABLE 4:** Statistics per Statement in Test Phase.

Statement	M	SD
I would like the same person to manage my applications over time so that I do not have to share my information multiple times	4.5	0.8
I would like to be supported with applications and/or filling out forms	3.9	0.8
It would be beneficial to me if different municipality departments can share my information (with my permission) so that I do not have to share this again myself	4.4	0.7
I would like to keep assistive technology for a longer or indefinite period of time, and that government employees pro‐actively think about what is useful and necessary	4.5	0.9
I would like government employees to pro‐actively offer suggestions about what we need to apply for and when, including matters outside of the WMO	4.7	0.6
I would like to receive a more extensive explanation about the WMO and as to why an application was denied	4.0	0.8
Everyone has the right to receive client support, and I would like to utilise this support, or at least be made aware of it	4.2	0.5
It would be helpful if the depreciation period for assistive technology is taken into account, and customised and appropriate solutions are offered	4.4	0.6
Should my application be denied, it would be nice to be offered alternatives, such as second‐hand assistive technology or sharing costs	4.3	0.7
I prefer to talk to, and have my applications be managed by someone who is knowledgeable about profound intellectual and multiple disabilities	4.9	0.4

The first text was rated high on both clarity (4.7) and expected increased understanding (4), as was the second text (4.2 and 4.0 respectively). Participants made minor textual suggestions for improvement that were incorporated. All 10 statements were rated highly as well (see Table 4 for the scores). The remarks by the participants did not reveal any major additional aspects that were important in relation to the management of WMO applications and the communication of government employees and the services they provide to warrant one or more additional statements, though several parents explained their scores or shared personal experiences. As both the text and statements were rated highly, an additional round of testing was not deemed necessary, and no further feedback from the participants was deemed to be required.

Based on the outcomes, an online training course (e‐learning), a common form of training for government employees, was developed. This course consisted of three short (between 210 and 280 words) introductory texts (one about persons with profound intellectual and multiple disabilities, one about their families, and one about why families applied for government support and how this was experienced). About each text two questions were asked to test the trainee's understanding about the subjects of the texts. This was followed by five fictional short (between 80 and 100 words) personal stories about conflicts or misunderstandings between parents and government employees, which the trainee was asked to provide a solution for, after which a possible solution was shown. The goal of engaging with these stories was to further increase the trainee's understanding of families with a child with profound intellectual and multiple disabilities and their experiences. At the end of the course, a summary of what was learned could be downloaded. Altogether, the training course was expected to take between 20 and 30 min to finish. The finished development of (the initial version of) the course also marked the end of the Design Thinking process, as the product to support families with a child with profound intellectual and multiple disabilities was developed per their needs and preferences.

## 3 Discussion

The aim of this study was to develop a product to support families with a child with profound intellectual and multiple disabilities. Through the method of Design Thinking, it was found that families found it important to be supported with strengthening their resilience and care capacity, and the preferred form would be a training course for government employees who manage WMO applications. Based on the feedback and input of parents, we were able to determine which elements should be included, which we used to develop an online training course, the goal of which was to improve the communication and mutual understanding between relatives of a person with profound intellectual and multiple disabilities and government employees.

The intermediate outcomes of the development process have provided additional and vital insights regarding the support of families with a child with profound intellectual and multiple disabilities. That is, while studies that have explored the support needs of these families have provided insights into what needs are present (e.g., Gauthier‐Boudreault et al. 2017; Jansen et al. 2013; Kruithof et al. 2022a; Lahaije et al. 2023a), they provide limited to no insights into the relative importance of needs, nor into the preferred content or form of the support. By engaging with the end‐users, as was done in this study, these insights can be gained, so that support may become more properly tailored. Notable in this regard are some of the outcomes of the Define phase. Although support from peers as well as family and friends is needed by parents (Lahaije et al. 2023a), parents assigned limited importance to improving contact with peers, and family, friends and so forth. This could mean these needs are already satisfied, parents already receive sufficient support, or do not require support as they are able to manage this need themselves. Meanwhile, improving contact with health care professionals and government employees was ranked considerably higher. Apart from differences in currently received support, these differences may also be explained in that there likely are differences in the roles and expectations of, (inter)dependency on, and required effort to effect and manage change in communicating with different (groups of) people, and the possible consequences of problems in communication. That is, while parents may not be significantly impacted by a misunderstanding with a friend and are able to manage and resolve this conflict themselves, resolving a conflict or effecting changes in communicating with a physician or government employee could be considerably harder, whilst also being heavily or completely dependent on their support (i.e., health care, financial and material aid), and possibly fearing retributions due to the power imbalance (Joseph‐Williams et al. 2014).

Notable also are the differences between the reported importance of the different kinds of informational support. While informational support regarding administrative matters and assistive technology (e.g., where and how to obtain it) were ranked highest in terms of importance, informational support regarding day care centres and residential facilities (e.g., where and which there are) was ranked in the lower half. Possible explanations are that parents already receive sufficient support regarding this subject, or that this type of information is more widespread, accessible and/or straightforward than information regarding administrative matters or assistive technology. Although more (qualitative) studies may be necessary to better understand which support needs are most important and why, the outcomes of the Define phase (and also of the Ideate phase) can already be informative to both practice and policy, and contribute to better attune support.

The outcomes of the Ideate phase show that parents prefer solutions aimed at adapting the environment and/or requiring the investment of others over solutions that require (significant) investment on their part. While adapting and transforming the socio‐ecological environment to accommodate families is a core theme within the contemporary view on disability and families (Thompson et al. 2017; Turnbull and Turnbull 2002), it is noteworthy that families themselves show a preference for this as well, and could therefore provide further incentive to do so. Several parents specifically indicated they did not have the resources to spend time on a training course (in line with the findings of previous studies that these families have limited leisure time [Geuze et al. 2022; Lahaije et al. 2023a, 2023b; Luijkx et al. 2017]), which should also inform and contribute to refining (the development of) future support and interventions for these families, in that these are sensitive and adapted to the preferences and circumstances of parents and families. Finally, it is of interest that the highest ranked and ultimately selected solution, namely communication training for government employees, (indirectly) incorporated some of the other problems that were ranked in the upper half in the Define phase, namely improving contact with government employees, and informational support regarding administrative matters and assistive technology. This suggests that the various support needs are interconnected, and that families could benefit from an integrative and systemic approach in practice and research, though more research is needed to understand the relationships between needs.

### 3.1 Limitations

The method of Design Thinking, used in this study to develop the product, is a method that is user‐centric and allows for creative solutions due to its open framework. However, this open framework and the fact that there is no ‘right’ way to utilise Design Thinking also mean, in comparison to a design method such as intervention mapping (a design framework for systematically developing theory‐ or practice‐based interventions; Bartholomew Eldredge et al. 2016), that it is more difficult to determine or verify if the development process was properly executed. Similarly, in contrast to intervention mapping (Bartholomew Eldredge et al. 2016), the solutions or products created through Design Thinking are not necessarily developed with theory‐ or evidence‐based methods. Such methods may still be used, though, or solutions may be grounded in theory otherwise. In this study, for example, several of the ideas or solutions were based on theory (e.g., strengthening family resilience by strengthening family communication, adapting the environment to accommodate families). Although the solutions created through Design Thinking may not necessarily be grounded in theory or evidence‐based methods, the flexibility of the open framework does allow for more creative or out‐of‐the‐box solutions, which can be especially useful and appropriate for problems that are difficult to solve (through conventional means), such as ill‐defined or complex problems (Dam and Siang 2018; Kumar et al. 2016).

For this study, the method of Design Thinking was adapted to the circumstances and intermediate outcomes of the study. Although this flexibility is part of the method, it may have had possible limitations. We opted to engage parents through online questionnaires, and not through, for example, focus groups. This method was selected because of the advantage that participants would be able to fill out the questionnaire at their convenience. A possible limitation is that parents could not be more directly and personally engaged with, nor could parents engage with each other, which could have provided additional insights. However, we believe that the selected method was preferable, as it likely allowed more parents to participate, and thus more insights could be gathered. Moreover, we had directly engaged with parents in previous studies (including an extensive qualitative study), the insights of which complemented and informed the (intermediate) outcomes of this study. Additionally, while Design Thinking can be an iterative process, we opted not to repeat any phases, as we found the outcomes to be clear and fairly unambiguous. However, the outcome of the Ideate phase showed a preference by the participants for solutions in which the socio‐ecological environment was adapted and/or required the investment of others. This phase could have therefore been repeated with ideas solely focused on adapting or transforming the environment, and ideas in which others had to make investments instead of families themselves. However, this does not imply that the developed product was not appropriate, as it aims to adapt (part of) the environment and matches the participants' preferences.

Participants in this study were recruited through convenience sampling. A disadvantage of this method is that it may lead to a sampling bias. While it is unknown whether and to what degree there is a sampling bias, and how this could have affected the outcomes of this study, a possible bias is that the calls and questionnaires were in Dutch, and therefore it was likely that only persons who spoke Dutch fluently participated. However, families with a child with profound intellectual and multiple disabilities who do not speak Dutch fluently may have (a different ranking of) needs, for example, in relation to securing government support, or to their communications with health care professionals. Similarly, because of convenience sampling, the number of hard‐to‐reach families and/or families with limited social capital included in this study may be limited, and their specific needs underrepresented. Likewise, no special effort was made to include participants from ethnic or religious minorities (nor were these background characteristics gathered), and their specific needs (if any) could similarly be underrepresented. Including the specific needs (if any) of these different groups could have led to adaptations or additions to the developed training course. Additionally, only parents were included in this study, not siblings or persons with profound intellectual and multiple disabilities. However, it is known that siblings have a somewhat different experience of FQOL (Lahaije et al. 2023b), and may therefore also have different perspectives on family support. Although it is unknown whether and how the outcomes of this study would change by including the perspectives of family members other than the parents, future studies may opt to include these perspectives to complement and/or enrich the perspectives of parents. Moreover, there may be discrepancies, tensions, or even conflicts in needs between parents and other family members, specifically the person with profound intellectual and multiple disabilities (e.g., sexual needs of the person with profound intellectual and multiple disabilities or their preferred residency). A proxy who is familiar with and can advocate on behalf of the person with profound intellectual and multiple disabilities may therefore be required. Finally, the studies that informed the Empathise phase all took place in a welfare state with social support (Nugroho 2018). While these were relevant for the current study (which took place in the Netherlands, a [conservative] welfare state [Nugroho 2018]), these studies would be less applicable for countries and cultures with less to no social support. Similarly, the online training course that was developed in this study is only applicable to the Netherlands, due to its relation to the WMO. However, while the intermediate outcomes (of the Define and Ideate phases) may not be applicable for countries with less to no social support, these outcomes could still be informative for other welfare states with social support similar to the Netherlands.

### 3.2 Future Directions

This study has shown that engaging parents with a child with profound intellectual and multiple disabilities in the product development leads to a product that matches their needs and also provides additional insights into their needs. Future product development studies for these families should similarly opt to involve parents, as it ensures that the form, content and type of product match their needs and preferences. Moreover, these studies could also include the perspectives of siblings and/or persons with profound intellectual and multiple disabilities and may also opt to further engage with family members by including them as co‐researchers. Furthermore, future studies may use a (more) representative sample of participants, for example, by making a special effort to include hard‐to‐reach families to ensure that the specific needs (if any) of these families (and other underrepresented and/or minority families) are heard and represented in the product development. Additionally, future studies may be informed by the insights of the Define and Ideate phases to understand which kind of support is preferred by families with a child with profound intellectual and multiple disabilities and develop support accordingly. Finally, future studies may, from the start of the development process, be informed by what parents have implicitly and explicitly stated in this study, namely that they prefer solutions aimed at adapting and transforming the socio‐ecological environment and/or require the investment of others over solutions that require (significant) investment on their part. A different approach in the development process could therefore be to begin by assessing what adaptations and transformations families with a child with profound intellectual and multiple disabilities prefer in order to experience the positive personal outcomes and well‐being they wish to experience and then continue developing a corresponding solution.

For a future implementation of the online training course created in this study, it is necessary to evaluate it, learn if it is effective and study how to implement it. Various models, tools (Attwell 2006), and success determinants (Al‐Fraihat et al. 2020) are available to specifically evaluate e‐learning, which should be used for an initial study. This evaluation may also include a qualitative study into the preferences of the government employees using the course, as well as their facilitators and barriers for change, which may then be incorporated into the course. Next, to understand if the course has the intended effect (namely to strengthen the communication and mutual understanding between relatives of a person with profound intellectual and multiple disabilities and government employees), a feasibility study (Orsmond and Cohn 2015) should be conducted. Ideally, both the experiences of the trainees and the relatives who were in contact with the trainees should be explored through a qualitative study to determine if the training course leads to improved communication and mutual understanding. The group of relatives should preferably include (ethnic and/or religious) minority and underrepresented groups, so as to ensure the course is inclusive and sensitive to the needs and preferences of minorities as well. Finally, to successfully implement the course, an implementation strategy, by applying for example implementation mapping (Fernandez et al. 2019), should be developed and applied. Such a strategy includes identifying the different stakeholders and their needs, the different conditions and requirements to implement the training course successfully, as well as an evaluation protocol. Of consideration in this regard could be that government employees who manage WMO applications are employed through different means, and the training course may therefore have to be implemented accordingly.

## 4 Conclusion

In this study, we aimed to develop a product to support families with a child with profound intellectual and multiple disabilities. Through the method of Design Thinking, we were able to develop an online training course for government employees, to strengthen their communication with and mutual understanding between them and relatives of a person with profound intellectual and multiple disabilities. In turn, this course is expected to strengthen the resilience and care capacity of families with a child with profound intellectual and multiple disabilities. This study has also provided additional insights into preferences of parents with a child with profound intellectual and multiple disabilities regarding their support.

## Author Contributions

Study design and set‐up was done by the first author with input from the co‐authors, data gathering and data analysis was done by the first author, writing of the manuscript was done by the first author with input from the co‐authors.

## Ethics Statement

Ethical permission for this study was granted by the ethical committee of the department of Pedagogical & Educational Sciences (Ethische Commissie PEDON) after independent review. Written informed consent was gathered from all participants.

## Conflicts of Interest

The authors declare no conflicts of interest.

## Data Availability

Research data are not shared.
